# Effects of topical application of 0.4% oxybuprocaine hydrochloride ophthalmic solution and 1% ropivacaine hydrochloride on corneal sensitivity in rats

**DOI:** 10.1371/journal.pone.0241567

**Published:** 2020-11-05

**Authors:** Cristina A. Lelescu, Andrada E. Urdă-Cîmpean, Daria A. Dumitraș, Marian Taulescu, Cosmin Mureșan

**Affiliations:** 1 VisionVet, San Giovanni in Persiceto, Bologna, Italy; 2 Department of Medical Informatics and Biostatistics, Faculty of Medicine, “Iuliu Hațieganu” University of Medicine and Pharmacy, Cluj-Napoca, Romania; 3 Department of Biochemistry, Faculty of Veterinary Medicine, University of Agricultural Sciences and Veterinary Medicine, Cluj-Napoca, Romania; 4 Department of Pathology, Faculty of Veterinary Medicine, University of Agricultural Sciences and Veterinary Medicine, Cluj-Napoca, Romania; 5 Department of Surgery, Faculty of Veterinary Medicine, University of Agricultural Sciences and Veterinary Medicine, Cluj-Napoca, Romania; University of Bari, ITALY

## Abstract

The aim of the present study was to determine and compare the degree and duration of corneal anesthesia following topical application of 0.4% oxybuprocaine hydrochloride ophthalmic solution and 1% ropivacaine hydrochloride treatment in healthy rats. A randomized, blinded, crossover study was conducted on 20 healthy adult Wistar rats, following complete physical and ophthalmological examination. Baseline corneal touch threshold (CTT) was determined in the central corneal area of both eyes with a Cochet–Bonnet aesthesiometer, in mm filament length. Oxybuprocaine was randomly applied to one eye and 0.9% sterile sodium chloride solution was instilled into the contralateral eye. Subsequent CTT measurements were performed in both eyes 5 minutes after topical application and at 5-minute intervals thereafter for 75-minutes in the anesthetized eye. Following a 2-week washout period, this protocol was repeated with ropivacaine. Quantitative data were summarized as mean ± standard deviation, median and inter-quartile range (Q1–Q3). Repeated measures data were analyzed over time and between treatments using Friedman test and Wilcoxon signed-rank test with Bonferroni adjustment (*p* < 0.05). Baseline CTT values were 60 mm in all eyes. With oxybuprocaine, CTT values decreased significantly for 65 minutes (0–55 mm; *p* = 0.002) when compared with baseline; the maximal anesthetic effect (no blink response at 5 mm filament length) was maintained for up to 15 minutes (*p* < 0.0001). With ropivacaine, CTT values were significantly lower than baseline for 30 minutes (0–55 mm; *p* = 0.002), with a maximal anesthetic effect recorded at 5 minutes in 18 eyes (*p* < 0.0001). Oxybuprocaine induced a significantly lower CTT than ropivacaine (*p* = 0.002) from 10 to 65 minutes following topical application. Both anesthetic agents induced significant corneal anesthesia; however, oxybuprocaine provided a greater and longer anesthetic effect, making it more suitable for potentially painful ophthalmologic procedures.

## Introduction

The rodent species are commonly used in biomedical research, due to the anatomical, physiological and genetic similarities they share with humans [1]. Moreover, the number of pet rodents has increased significantly in recent years, which has led to an increase in the demand for veterinary healthcare services for this species [2].

Many diagnostic and therapeutic procedures in veterinary ophthalmology such as corneal and conjunctival scrapings, corneal suture, tonometry, intracameral injections, and foreign body extractions are performed under topical anesthesia [3]. Various local anesthetics such as proparacaine, tetracaine and oxybuprocaine are commonly used in veterinary ophthalmology to desensitize the cornea [3–6].

Oxybuprocaine (benoxinate) is a para-amino-benzoic acid ester, initially used in human ophthalmology as a topical anesthetic for minor procedures of the cornea [7]. Oxybuprocaine hydrochloride 0.4% ophthalmic solution is routinely used in Europe for topical anesthesia of the cornea in both large and small animals [5,8], and its efficacy has been assessed in dogs [5], cats [9], equids [10] and cattle [11].

Ropivacaine hydrochloride is the S-enantiomer of N-propyl pipecolyl xyilidine [12]. In humans, topical administration of ropivacaine resulted in effective and persistent anesthesia during cataract surgery, which virtually eliminated the need for intracameral anesthesia supplementation [13].

The Cochet-Bonnet aesthesiometer is widely used to quantitatively assess corneal sensitivity in both humans [14] and animals [15,16]. The measurement is performed by means of a nylon filament, which has an adjustable length between 5 and 60 mm. According to von Frey’s principle, the longer the length of the filament, the lower the pressure it exerts [5]. Pressure is exerted by application of the filament perpendicular to the cornea until it bends. The applied pressures vary between 5 to 180 mg S^-1^, where S = 0.0113 mm^2^ (0.4–15.9 g·mm^2^). The pressure required to evoke a blinking reflex is defined as the corneal touch threshold (CTT), which is the most commonly determined variable [6].

Although many studies have investigated the local anesthetic activity of 0.4% oxybuprocaine hydrochloride solution on corneal sensitivity in a variety of animal species, none have addressed its efficacy and duration of action following topical administration in rats. Furthermore, the efficacy of 1% ropivacaine hydrochloride for topical anesthesia of the cornea is little documented in animals, particularly in rats. In this context, the aim of our study was to evaluate and compare the efficacy and duration of topical 0.4% oxybuprocaine hydrochloride ophthalmic solution and 1% ropivacaine hydrochloride on corneal sensitivity in clinically healthy rats. Our hypothesis was that oxybuprocaine would provide significant corneal anesthesia and that ropivacaine would have a comparable anesthetic effect.

## Materials and methods

### Animals

A group of 20 Wistar albino rats (*Rattus norvegicus*) weighing approximately 350 grams, of both sexes (12 females and 8 males), were used in this study. The inclusion criteria were: adult rats that were declared healthy following a complete clinical and ophthalmological examination. The sample size was deduced from previous similar studies, which showed a rather heterogeneous sampling size: i.e. 8 [beagles, [17]], 9–10 [rats, dogs, [16]], 12+12 [cows, [11]], 12–22 [dogs, [5]], 18 [cats, [9]], 20 [cats, [18]]. In addition, this sample size resulted in an attempt to conform with the guiding principles for the ethical use of animals in testing ("The Three Rs"). The animals were obtained from an authorized breeding unit and were housed according to ISO 10993–2 (ISO 10993–2, 2004) in individual ventilated cages, at 25 ± 2°C and 55% ± 10% relative humidity, under a 12 hours light-dark cycle, in the authorized University Laboratory Animal Facility (University of Agricultural Sciences and Veterinary Medicine Cluj-Napoca, Romania). Standard rodent diet (Cantacuzino National Institute of Research, Romania) and water *ad libitum* were provided. All animal procedures were approved by the Bioethics Committee of the University of Agricultural Sciences and Veterinary Medicine Cluj-Napoca (no. 129/20.12.2018) and followed the guidelines of the European Law Directive 63/2010, materialized by Romanian Law no. 43/2014.

### Study design

A randomized, blinded, crossover study was conducted. Baseline corneal touch threshold (CTT) was measured bilaterally in the central corneal area of all rats, with a Cochet–Bonnet aesthesiometer, in mm filament length. Subsequently, 3 drops (50 uL drop^-1^) of 0.4% oxybuprocaine hydrochloride (Benoxi; Unimed Pharma Ltd, Slovakia) were instilled within 3 minutes (1 drop minute^-1^) into one randomly selected eye [19], and an equivalent volume of 0.9% sterile sodium chloride solution (NaCl 0.9%; BBraun Melsungen AG, Germany) was concomitantly applied to the contralateral eye. CTTs were measured in both eyes 5 minutes after topical application and at 5-minute intervals during the subsequent 75-minute period for the anesthetized eye. After a washout period of 14 days, 1% ropivacaine hydrochloride (Ropivacaina Kabi; Fresenius Kabi Norge AS, Norway) was topically administered to the same (previously tested) eye, according to the abovementioned protocol (3 drops within 3 minutes; 50 uL drop^-1^). Both oxybuprocaine and ropivacaine were administered by the same person, ensuring that all administered drops entered the palpebral fissure (manually opened) and remained on the ocular surface for 1 second. This person was not blinded to the order of the treatment regimen. The CTT measurements were performed in the same examination room by one evaluator, unaware of which eye was treated with the anesthetic agent. Furthermore, handling of the rats was conducted by an experienced team member; the animals were restrained in a comfortable standing position, with minimal restraint to the head and eyelids and without compressing the jugular veins, in order to minimize the effect of restraint on the data we obtained.

Any symptom of ocular irritation following topical instillation of the local anesthetic solutions, such as blepharospasm, conjunctival hyperemia, chemosis, itching, epiphora and eyelid inflammation was recorded as “present” or “absent”. None of the rats were euthanized during or at the end of the study.

### CTT measurements

A Cochet-Bonnet esthesiometer (Western Ophthalmics, WA, USA) was used to measure CTTs throughout the study. The nylon filament (0.12 mm diameter) extended to its maximum length (60 mm) was applied perpendicularly to the central cornea of the rats, and advanced until it bent slightly, in accordance with a previous study [16]. When no blink reflex was obtained in a minimum of 3 out of 5 stimulations, the filament was shortened by 5 mm and the measurement was repeated (at the same timepoint) until more than half of the stimulations resulted in a blink response. Consequently, the reported CTT value was the shortest length of the nylon filament (in mm) that caused a blink reflex in at least 3 out of 5 stimulations [9,16].

### Statistical analysis

Counts and percentages were used to quantify how many rats regained corneal sensitivity at a given time. Quantitative data were summarized as mean ± standard deviation (SD) for normally distributed data or median and inter-quartile range [IQR or Q1 − Q3, where Q1 = the 25th percentile and Q3 = the 75th percentile)] for non-parametric data. Since Shapiro-Wilk test showed that the CTT values were not normally distributed, Friedman test (with Bonferroni adjustment) and Wilcoxon signed-rank test were used to compare CTT values for repeated measurements over time and between treatments. Statistical analysis was performed using SPSS Statistics software, version 25 (IBM Corporation, NY, USA). According to the number of comparisons, the correction set the statistical significance level at a *p* -value of 0.0033 for different measurements of the same treatment and 0.0031 for oxybuprocaine *versus* ropivacaine treatments.

## Results

The baseline CTT values measured in both eyes of all rats (40 eyes) were 60 mm. Both drugs showed a rapid onset of action, and the maximum anesthetic effect was achieved within 5 minutes in all 20 oxybuprocaine treated eyes and in 18 out of 20 ropivacaine-treated eyes. Corneal sensitivity remained unchanged in all 20 eyes at 5 minutes following topical instillation of 0.9% saline.

**Fig 1:**
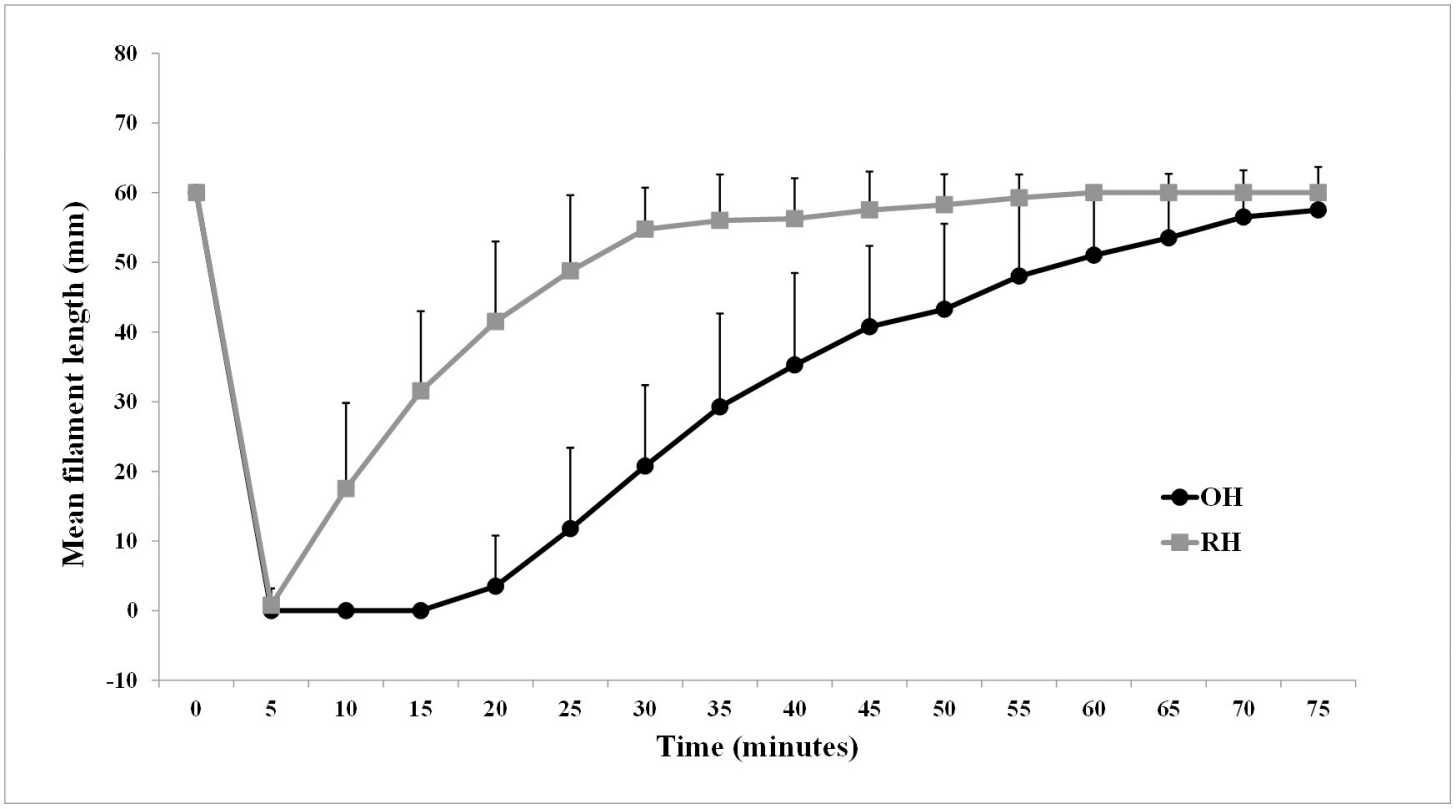
Comparison of mean corneal touch threshold (CTT) values in mm filament length (± standard deviation) obtained at baseline and at 5-minute intervals thereafter, for a total of 75 minutes following application of topical 0.4% oxybuprocaine hydrochloride ophthalmic solution (OH) and 1% ropivacaine hydrochloride (RH) in clinically healthy rats. The CTT in mm is further converted to pressure in grams per mm^2^ and in milligrams per S (S = 0.0113 mm^2^ sectional area of the filament), according to the conversion table provided by the manufacturer.

In the oxybuprocaine-treated eyes, CTT values were significantly decreased during the first 65 minutes (varying from 0 to 55 mm; *p* = 0.0029) following topical application, compared with the baseline values (Fig 1). The maximal anesthetic effect, equivalent to a CTT value of 0 (no blink response at a filament length of 5 mm), was accomplished within 5 minutes after topical application of oxybuprocaine and lasted for 15 minutes in all 20 eyes (Table 1). Duration of complete corneal anesthesia ranged between 15 and 35 minutes, while CTT values returned to baseline within 45 to 75 minutes in all but 3 oxybuprocaine—treated eyes.

**Table 1:** Summary of corneal touch threshold (CTT) measurements obtained before and after topical application of 0.4% oxybuprocaine hydrochloride (OH) ophthalmic solution or 1% ropivacaine hydrochloride (RH) in clinically healthy rats.

Variable	OH- treated eyes (*n* = 20)	RH- treated eyes (*n* = 20)
**Baseline CTT (mm) or [g/mm**^**2**^**]**	60 [0.4]	60 [0.4]
**Onset of action (minutes)**	5 or less	5 or less
**Minimum CTT achieved (mm) or [g/mm**^**2**^**]**	0 [> 15.9]	0 [> 15.9] (*n* = 18)
**Time to achieve minimum CTT (minutes)**	5 or less	5 or less (*n* = 18)
**Duration of the maximal anesthetic effect (CTT = 0), (minutes)**	15	5 (*n* = 18)
**Duration of topical anesthesia (minutes)**	65*	30*

Pressures are shown as filament length (mm) or applied pressure (g/mm^2^), where a filament length equal to 5 mm corresponds to a pressure greater than 15.9 g/mm^2^. A total of 20 animals were studied and *n* represents the number of eyes treated unless indicated in the relevant column. Onset of action, time to achieve minimum CTT, duration of maximal anesthetic effect and duration of topical anesthesia are shown. Duration of topical anesthesia was measured by comparing the mean CTT values obtained every 5 minutes following drug administration with mean baseline values and including the last measurement in which a statistically significant difference was still present (CTT varied from 0 to 55 mm; *p* = 0.0029).

* CTT varied from 0 to 55 mm; *p* = 0.0029.

In the ropivacaine-treated eyes, CTT values were significantly lower than baseline measurements during the first 30 minutes following topical anesthesia (varying from 0 to 55 mm; *p* = 0.0029), (Fig 1). Moreover, a maximal anesthetic effect was achieved in 18 treated eyes within 5 minutes following topical application of ropivacaine and lasted for 5 minutes (Table 1). In treated eye of two rats, complete anesthesia was not achieved at any timepoint. CTT values returned to baseline within 20 to 60 minutes, in all 20 ropivacaine-treated eyes.

Oxybuprocaine-treated eyes had significantly lower CTT values than those treated with ropivacaine for a period of time ranging from 10 to 65 minutes after topical administration (Wilcoxon signed ranks test: *p* = 0.0029). Data are summarized in Table 2 using descriptive statistics including mean (± SD), median and interquartile range (IQR).

**Table 2:** Descriptive statistics including mean ± standard deviation (SD), median and interquartile range (IQR) of CTT values (mm) obtained at baseline and at 5-minute intervals thereafter, for a total of 75 minutes following application of topical 0.4% oxybuprocaine hydrochloride ophthalmic solution (OH) and 1% ropivacaine hydrochloride (RH) in clinically healthy rats.

Minutes after topical drug administration	Mean CTT (SD), (mm)		Median CTT (IQR), (mm)	
	OH	RH	OH	RH
**Baseline**	60 (± 0)	60 (± 0)	60 (60–60)	60 (60–60)
**5**	0 (± 0) †	1 (± 2) †	0 (0–0)	0 (0–0)
**10**	0 (± 0) †	18 (± 12) †*	0 (0–0)	20 (6–29)
**15**	0 (± 0) †	32 (± 11) †*	0 (0–0)	35 (20–40)
**20**	4 (± 7) †	42 (± 11) †*	0 (0–4)	40 (36–50)
**25**	12 (± 12) †	49 (± 11) †*	10 (0–20)	50 (40–60)
**30**	21 (± 12) †	55 (± 6) †*	20 (10–30)	55 (50–60)
**35**	29 (± 13) †	56 (± 7) *	30 (18–35)	60 (55–60)
**40**	35 (± 13) †	56 (± 6) *	35 (26–45)	60 (50–60)
**45**	41 (± 12) †	58 (± 6) *	38 (35–54)	60 (60–60)
**50**	43 (± 12) †	58 (± 4) *	43 (35–55)	60 (60–60)
**55**	48 (± 12) †	59 (± 3) *	50 (40–60)	60 (60–60)
**60**	51 (± 10) †	60 (± 0) *	53 (46–60)	60 (60–60)
**65**	54 (± 9) †	60 (± 0) *	55 (50–60)	60 (60–60)
**70**	57 (± 7)	60 (± 0)	60 (55–60)	60 (60–60)
**75**	58 (± 6)	60 (± 0)	60 (60–60)	60 (60–60)

Descriptive statistics are shown as whole numbers.

* significantly different from oxybuprocaine, p = 0.0029.

^†^ Significantly different from baseline, p = 0.0029.

No symptoms of ocular irritability were observed in any of the rats during the 75-minute evaluation interval, following topical administration of either oxybuprocaine or ropivacaine.

## Discussion

The main findings of this study suggest that topical administration of 0.4% oxybuprocaine hydrochloride and 1% ropivacaine hydrochloride induce significant corneal anesthesia in rats. Furthermore, it was shown that oxybuprocaine hydrochloride causes a more effective and longer acting corneal anesthesia than ropivacaine hydrochloride (65 minutes *versus* 30 minutes). Complete corneal anesthesia (CTT = 0) was achieved by 5 minutes after application of oxybuprocaine hydrochloride to all 20 eyes, and lasted for 15 minutes, while application of ropivacaine hydrochloride induced complete corneal anesthesia within 5 minutes in 18 out of 20 eyes and lasted for only 5 minutes.

Baseline CTTs were 60 mm in all eyes, which is equivalent to 0.4 g/ mm^2^. This value is considerably higher than that reported by Kim et al., 2013, which is to be expected since a low dose of a tiletamine/zolazepam combination was used for sedation of rats prior to CTT measurements. Conversely, considerably lower baseline CTT values were obtained in a group of Sprague-Dawley rats [20]. Although certain variations in corneal sensitivity within strains may be possible, this discrepancy is more likely to be caused by differences in handling of the aesthesiometer and interpretation of the corneal blinking reflex between evaluators.

Oxybuprocaine hydrochloride is widely used as a topical anesthetic for diagnostic and surgical procedures in human and veterinary ophthalmology in most European countries [21,22], due to a lower occurrence of adverse effects compared to other drugs (*e*.*g*. tetracaine) [5,10], low risk of serious adverse effects [23] (and no interference with the precorneal tear film stability [24]. At present, a 0.4% oxybuprocaine hydrochloride ophthalmic solution is commercially available. To the authors’ knowledge, no previous studies were conducted in laboratory rodents. However, in dogs and cats, corneal sensitivity was found to be significantly decreased for 45 minutes following topical administration [5,9]. While the present study showed that a similar magnitude of effect in rats occurred for 65 minutes. This difference may be attributed to the fact that rats have a smaller corneal surface area than dogs and cats, while the volume of a single drop instilled into the eye is identical. Moreover, the number of drops instilled into each eye was higher in our study (3 drops instead of a single drop), since about one-third of the rats moved their heads immediately after dispensing the first drop, regardless of which treatment (drug or 0.9% sodium chloride) was applied. This behavior could not be controlled under minimal restraint conditions used in this study.

Complete corneal anesthesia was observed in all oxybuprocaine-treated eyes within 5 minutes after instillation and persisted 15 minutes; similar results were obtained in dogs [5], whereas in cats complete anesthesia of the cornea persisted only for 5 minutes [9]. It should be noted that the first CTT measurement was performed 5 minutes after drug instillation in our study, and therefore the onset of complete corneal anesthesia may have occurred sooner. In dogs, cats and cattle, no blink reflex was noted in response to the shortest filament length from the first minute after topical administration [5,9,11]. No symptoms of ocular irritation were observed in rats with this ophthalmic formulation of oxybuprocaine, which agrees with these aforementioned studies [5,9,11].

The clinical use of ropivacaine hydrochloride has increased in human anesthesia and analgesia practice in recent years [25]. While, several studies have addressed the clinical utility of ropivacaine hydrochloride in human and veterinary ophthalmology [26–30]. Most of these research studies assessed the effectiveness of ropivacaine for peribulbar anesthesia and as a topical agent in cataract surgery. Few studies have investigated its effect on corneal sensitivity [17,31], and, to the author’s knowledge, no similar study was performed out in rats.

The corneal sensitivity in the ropivacaine-treated rats was decreased significantly during the first 30 minutes following topical application, which is slightly longer than in dogs given a single 50 μg drop^-1^ dose [17]. The possible causes for this inconsistency are similar to those aforementioned for oxybuprocaine (smaller corneal surface area than dogs, 3 drops instilled into each eye *versus* a single drop). Moreover, it has been demonstrated that topical administration of 2 drops of ropivacaine to rabbits resulted in effective and significantly longer anesthesia than instillation of a single drop [31]. In addition, corneal aesthesiometry is a subjective evaluation, influenced by numerous factors including species, age and shape of the individual’s skull, ambient humidity and temperature [6]. Even though topical ropivacaine induces anesthesia of sufficient magnitude and duration for short ophthalmologic procedures in both humans and dogs [17,27]; our results suggest that it provides an incomplete and limited anesthetic effect in rats. CTT measurements performed 10 minutes after ropivacaine hydrochloride instillation showed variations in filament length (applied pressure) ranging between 0 to 35 mm. Therefore, the authors believe that these variations in corneal sensitivity are too large to recommend its topical use for surgical procedures in rats. Still, it may be suitable for short diagnostic procedures such as corneoconjunctival cytology, applanation tonometry, gonioscopy, ultrasonography, which can be completed in less than 10 minutes but may cause some discomfort.

Topical administration of ropivacaine was well tolerated by all rats and no signs of ocular irritability were observed during the examination. These findings are consistent with those reported in human patients and rabbits [27,31], although mild conjunctival hyperaemia, blepharospasm, and lacrimation were observed in dogs following ropivacaine instillation [17].

Our study has several limitations. First, CTT measurements were performed in the sodium chloride-treated eyes only once, 5 minutes following instillation, to minimize the stressful effect of this procedure. Repeated exposure to an unpleasant procedure may have increased the anxiety of the rats, which is undesirable when measurements are performed without sedation and with minimal physical restraint. In addition, the small size of the rat’s eye, rapid motion and high agility of this species may affect the accuracy of the measurements. This is particularly so during the gradual fading of the anesthetic effect, when changes in corneal sensitivity between two consecutive measurements may be uncertain. Subjective perception of the filament deflection by the evaluator is a potential limitation when using the Cochet-Bonnet aesthesiometer [18]. However, in our study, use of the aesthesiometer and interpretation of the corneal response to filament stimulation was performed by the same evaluator in all rats and the procedure was performed in a temperature and humidity-controlled room, to minimize variability in the filament rigidity. Also, the onset of complete corneal anesthesia may have occurred sooner, owing to the fact that the first evaluation was performed at 5 minutes following drug instillation. In addition, administration of three drops instead of one could affect the duration of action and the duration of peak action. Another consideration is the potential for systemic toxicity of topical anesthetics. It is reported that following topical application of a drug, less than 10% of the administered dose traverses the cornea, sclera and conjunctiva and enters the eye itself.; while approximately 50% is absorbed systemically via the conjunctiva and naso-lacrimal drainage system [32]. This may increase the risk of overdose and additional caution is required when applying topical medication, especially in small animals such as rats. In addition, in a clinical setting, the animal may have variable reactions to administration (such as head shaking, blinking, and eye rubbing), and repeated doses are needed. Also, given the dosage instructions listed in the oxybuprocaine package leaflet for ocular anesthesia in humans (1 drop minute^-1^, 3 times), potential toxicity was a major one of our concerns. However, none of the subjects manifested signs of systemic toxicity and according to our calculations, the total administered dose was considerably lower than the reported median lethal dose (LD_50_) of both drugs following intravenous injection in rats [33,34]. We propose that the topical application of oxybuprocaine hydrochloride and ropivacaine hydrochloride according to our abovementioned protocol may be considered for subsequent investigations.

In conclusion, we believe this study is the first to determine the efficacy of topical oxybuprocaine hydrochloride and ropivacaine hydrochloride for corneal anesthesia in rats. These findings suggest that both anesthetic agents may induce significant corneal anesthesia; however, oxybuprocaine hydrochloride ophthalmic solution provides a greater and longer anesthetic effect, which makes it a more suitable agent for potentially invasive ophthalmologic procedures.

## Supporting information

S1 Table Descriptive statistics including mean, standard deviation (SD), median and interquartile range (IQR) of corneal touch threshold (CTT) values (mm) obtained at baseline and at 5-minute intervals thereafter, for a total of 75 minutes following application of topical 0.4% oxybuprocaine hydrochloride ophthalmic solution (OH) and 1% ropivacaine hydrochloride (RH). Descriptive statistics are shown with two decimal places.
(DOCX)
