# Supplementary material for: Effects of topical application of 0.4% oxybuprocaine hydrochloride ophthalmic solution and 1% ropivacaine hydrochloride on corneal sensitivity in rats
Source: PLoS One. 2020 Nov 5;15(11):e0241567. doi: 10.1371/journal.pone.0241567 (PMC7644035; doi:10.1371/journal.pone.0241567)
Supplement: S1 Table — Descriptive statistics are shown with two decimal places. (DOCX) [file pone.0241567.s001.docx]

**Table 2.** Descriptive statistics including mean, standard deviation (SD), median and interquartile range (IQR) of corneal touch threshold (CTT) values (mm) obtained at baseline and at 5-minute intervals thereafter, for a total of 75 minutes following application of topical 0.4% oxybuprocaine hydrochloride ophthalmic solution (OH) and 1% ropivacaine hydrochloride (RH). **Descriptive statistics are shown with two decimal places.**

| **Minutes after topical administration** | **Mean CTT (SD), (mm)** | | **Median CTT (IQR), (mm)** | |
| --- | --- | --- | --- | --- |
|  | **OH** | **RH** | **OH** | **RH** |
| **Baseline** | 60.00 (0.00) | 60.00 (0.00) | 60.00  (60.00-60.00) | 60.00  (60.00-60.00) |
| **5** | 0.00 (0.00) † | 0.75 (2.44) † | 0.00  (0.00-0.00) | 0.00  (0.00-0.00) |
| **10** | 0.00 (0.00) † | 17.50 (12.30) †* | 0.00  (0.00-0.00) | 20.00  (6.25-28.75) |
| **15** | 0.00 (0.00) † | 31.50 (11.48) †* | 0.00  (0.00-0.00) | 35.00  (20.00-40.00) |
| **20** | 3.50 (7.27) † | 41.50 (11.48) †* | 0.00 (0.00-3.75) | 40.00  (36.25-50.00) |
| **25** | 11.75 (11.61) † | 48.75 (10.86) †* | 10.00  (0.00-20.00) | 50.00  (40.00-60.00) |
| **30** | 20.75 (11.61) † | 54.75 (5.95) †* | 20.00  (10.00-30.00) | 55.00  (50.00-60.00) |
| **35** | 29.25 (13.40) † | 56.00 (6.60) * | 30.00  (17.50-35.00) | 60.00  (55.00-60.00) |
| **40** | 35.25 (13.22) † | 56.25 (5.82) * | 35.00  (26.25-45.00) | 60.00  (50.00-60.00) |
| **45** | 40.75 (11.61) † | 57.50 (5.50) * | 37.50  (35.00-53.75) | 60.00  (60.00-60.00) |
| **50** | 43.25 (12.27) † | 58.25 (4.37) * | 42.50  (35.00-55.00) | 60.00  (60.00-60.00) |
| **55** | 48.00 (11.74) † | 59.25 (3.35) * | 50.00  (40.00-60.00) | 60.00  (60.00-60.00) |
| **60** | 51.00 (9.54) † | 60.00 (0.00) * | 52.50  (46.25-60.00) | 60.00  (60.00-60.00) |
| **65** | 53.50 (9.19) † | 60.00 (0.00) * | 55.00  (50.00-60.00) | 60.00  (60.00-60.00) |
| **70** | 56.50 (6.70) | 60.00 (0.00) | 60.00  (55.00-60.00) | 60.00  (60.00-60.00) |
| **75** | 57.50 (6.17) | 60.00 (0.00) | 60.00  (60.00-60.00) | 60.00  (60.00-60.00) |

* significantly different from oxybuprocaine, p = 0.0029

† Significantly different from baseline, p = 0.0029.
